# Comparison of Different Carbon Dioxide Insufflation Rates on Hemodynamic Changes in Laparoscopic Surgeries: A Randomized Controlled Trial

**DOI:** 10.7759/cureus.34071

**Published:** 2023-01-22

**Authors:** Rajagopalan Venkatraman, Tharun Ganapathy Chitrambalam, Anandpandi Preethi

**Affiliations:** 1 Anaesthesiology, SRM Institute of Science and Technology, Chennai, IND; 2 General Surgery, SRM Institute of Science and Technology, Chennai, IND

**Keywords:** shoulder pain, pneumoperitoneum, laparoscopic surgeries, hemodynamic, co2 insufflation

## Abstract

Introduction

The injury and detrimental effects of carbon dioxide (CO2) insufflation during laparoscopic surgeries may be due to the higher flow rates used during insufflation. The aim of our study was to study the effects of different CO2 insufflation flow rates on hemodynamic parameters in laparoscopic surgeries. The secondary objectives were to compare the patient and surgeon satisfaction scores, postoperative shoulder scores, and surgical site pain scores.

Methods

This prospective, randomized, double-blinded trial was commenced after institutional ethical committee approval and The Clinical Trials Registry- India (CTRI) registration (CTRI 2021/10/037595). Ninety patients scheduled for laparoscopic cholecystectomy were randomly divided into three groups based on CO2 insufflation flow rate by computer-generated random numbers and the sealed envelope method: Group-A: 5 L/min; Group-B: 10 L/min; and Group-C: 15 L/min. General anesthesia was standardized in all three groups. Mean arterial pressure (MAP) and heart rate were recorded at different timelines, which included the arrival in the operating room (T0), just before the induction of anesthesia (T1), at the beginning of pneumoperitoneum (T2), 10 minutes (T3), 20 minutes (T4), 30 minutes (T5), and 60 minutes (T6) after the pneumoperitoneum, at the end of the operation (T7), five minutes (T8), and 15 minutes (T9) after arriving at the recovery room. The patient and surgeon satisfaction scores were assessed on a 5-point Likert scale. The visual analog score (VAS) was used to assess the surgical site pain and shoulder pain every four hours for 24 hours. The continuous data were assessed by one-way analysis of variance (ANOVA), and the categorical data were assessed by the Chi-square test. The sample size was estimated based on a pilot study and using the G Power 3.1.9.2 Program (Universitat Kiel, Germany) calculator.

Results

There was an increase in the mean arterial pressure (MAP) between the groups 60 min after pneumoperitoneum creation with higher flow rates. The baseline MAP was 85.76± 10.11 in group A, 86.03± 9.79 in group B, and 88.13± 8.46 in group C. At 60 min from the creation of the pneumoperitoneum, the MAP increased significantly from 99.17 ± 9.35 in group A, 102.43 ± 8.24 in group B, to 106.83 ± 8.31 in group C. This was statistically significant with a p-value of 0.004. There was a statistically significant difference in heart rate between the groups 10 minutes after pneumoperitoneum creation. No complications were reported in any of the groups. The postoperative shoulder pain was more severe when higher flows were used at 20 and 24 hours. The surgical site pain was also significantly more for up to 12 hours following surgery with higher flows.

Conclusion

We conclude that low-flow CO2 insufflation during laparoscopic surgeries is associated with fewer hemodynamic changes, better patient satisfaction scores, and lower postoperative pain scores.

## Introduction

Laparoscopic surgeries have become the gold standard in the management of many gastrointestinal, urological, and gynecological surgeries [1]. They provide multiple advantages, like smaller incision sizes, lesser trauma, and faster recovery rates. better postoperative comfort and fewer postoperative wound infections with better cosmetic results [2-4].

The creation of carbon dioxide (CO2) pneumoperitoneum may result in increased blood pressure [5]. The hemodynamic changes include increased preload and afterload and decreased cardiac output. These hemodynamic insults may be severe in old age, in cardiac patients, or in patients with other systemic disorders and may even be life-threatening [6]. This is due to the absorption of CO2 from the peritoneal cavity into the bloodstream, resulting in hypercarbia. Hypercarbia can cause increased systemic vascular resistance and reduced left ventricular function with negative inotropic action on the heart [7]. The mechanical compression or tamponade effect of pneumoperitoneum on great vessels may decrease the preload, leading to reduced cardiac output.

We hypothesized that a low CO2 insufflation flow rate would result in fewer hemodynamic disturbances. We could not find any studies available comparing CO2 insufflation flow rates and blood pressure changes in the literature. Hence, we decided to study the different carbon dioxide insufflation rates and hemodynamic changes in laparoscopic surgeries. The primary objective was to study the changes in mean arterial pressure with different CO2 insufflation flow rates in laparoscopic cholecystectomy. The secondary objectives were to compare changes in heart rate, surgeon and patient satisfaction scores, postoperative shoulder pain, and surgical site pain postoperatively for 24 hours.

## Materials and methods

This double-blinded, randomized, controlled trial was done prospectively after Institutional Ethical Committee approval and Clinical Trials Registry-India (CTRI/2021/10/037595) registration. The patients in the age group between 18 and 60 years with a body mass index (BMI) between 18.5 and 24.99 and belonging to the American Society of Anesthesiologists (ASA) physical status I or II scheduled for elective laparoscopic cholecystectomy were incorporated in the study. Patients with hypertension, cardiac, renal, or liver diseases, pregnancy, or those who refused to participate in the study were excluded from the study. All the patients enrolled in the study gave written, informed consent.

Ninety patients scheduled for laparoscopic cholecystectomy were randomly divided into three groups: Group A: CO2 insufflation flow rate of 5 liters/minute (L/min); Group B: CO2 insufflation flow rate of 10 L/min; and Group C: CO2 insufflation flow rate of 15 L/min. The computer-generated random number table was used for the randomization of patients. The block randomization was done with random block sizes of four. To ensure allocation concealment, patients were allotted a unique number, written on a piece of paper with the group allocated, and placed inside an opaque envelope and sealed. The envelopes enclosed with the study group allocation were opened on the day of the surgery by an individual not associated with the study. The person opening the envelope sets the CO2 insufflation flow rate according to the group involved and takes no further part in the study. The surgeon and the anesthesiologist were blinded to the flow rate used during surgery.

All the patients received alprazolam 0.25 mg, ranitidine 150 mg, and metoclopramide 10 mg orally on the previous night and two hours before surgery. The intraoperative monitoring used included non-invasive blood pressure, a pulse oximeter, an electrocardiograph, capnography, and temperature. The mean arterial pressure (MAP) and heart rate were recorded from the time of arrival in the operating room (T0), just before the induction of anesthesia (T1), at the beginning of pneumoperitoneum (T2), 10 minutes later (T3), 20 minutes later (T4), 30 minutes later (T5), and 60 minutes later (T6) after the pneumoperitoneum, at the end of the operation (T7), and five minutes later (T8) and 15 minutes later (T9) after arriving at the recovery room. General anesthesia was standardized in all the groups and included glycopyrrolate 0.2 mg as an anti-sialagogue, fentanyl 2 mcg/kg as an intraoperative analgesic, propofol 2 mg/kg as an induction agent, and vecuronium 0.1 mg/kg for intubation and one-fifth of the dose for maintenance. The patients were intubated with appropriate endotracheal tube sizes, and anesthesia was maintained with oxygen, nitrous oxide, and sevoflurane.

Under the Veress needle insufflation technique, a pneumoperitoneum is created, and intraperitoneal pressure is set to 15 mm of Hg with flow rates set to 5 L/min, 10 L/min, and 15 L/min in Groups A, B, and C, respectively. Following port placement, the patient was placed in a 30° reverse Trendelenburg and 20° left lateral position, and the procedure commenced. The hemodynamic changes throughout the entire surgery were recorded and analyzed. No alterations were made to the pressure or flow rate throughout the procedure. In case of any hemodynamic instability during the procedure or any situation warranting manipulation of the pressure and flow rates, the subjects were withdrawn from the study. They were considered failures and recorded. The surgery proceeded at the flow rate needed by the surgeon.

A single, experienced surgeon performed all the surgeries. If there was an increase in mean arterial pressure of 20%, nitroglycerin (NTG) intravenous (IV) infusion was initiated with a dose of 0.05 mcg/kg and titrated accordingly. The patient was extubated at the end of the surgery, after reversal with neostigmine and glycopyrrolate, and shifted to the recovery room. The patients received paracetamol 1g IV before extubation and sixth hourly for 24h. The pain at the surgical site and shoulder pain were assessed using the Visual Analog Scale (VAS), with 0 being no pain and 10 being the worst possible pain [8]. If VAS > 6, tramadol 100 mg IV was administered along with ondansetron 4 mg IV. The total consumption of analgesics was recorded. The surgeon and patient satisfaction scores were recorded on a five-point Likert scale: five = excellent, four = adequate, three = cannot say, two = inadequate, and one = poor [9].

A G Power 3.1.9.2 program (Universitat Kiel, Germany) calculator was used for sample size estimation. We conducted a pilot study of 15 patients and obtained a mean MAP of 86.61, 87.6, and 87.8 in groups A, B, and C, respectively. Assuming a 95% significance level and 95% power, we obtained a sample size of 85. The total sample size was taken as 90, assuming 5% dropouts. Patients from the pilot study were not included in the final analysis. The data were entered into an MS Excel spreadsheet (2021) and were analyzed using the statistical package for social sciences version 22 (trial version). Descriptive statistics, including proportions, measures of central tendency, and measures of dispersion, were used to describe the data. The Skewness-Kurtosis All test were used to determine the normalcy of the distribution. The categorical variables were analyzed using the Chi-square test. The comparison of continuous variables was carried out by the one-way analysis of variance (ANOVA) test. A p-value of < 0.05 was considered to be statistically significant.

## Results

**Figure 1:**
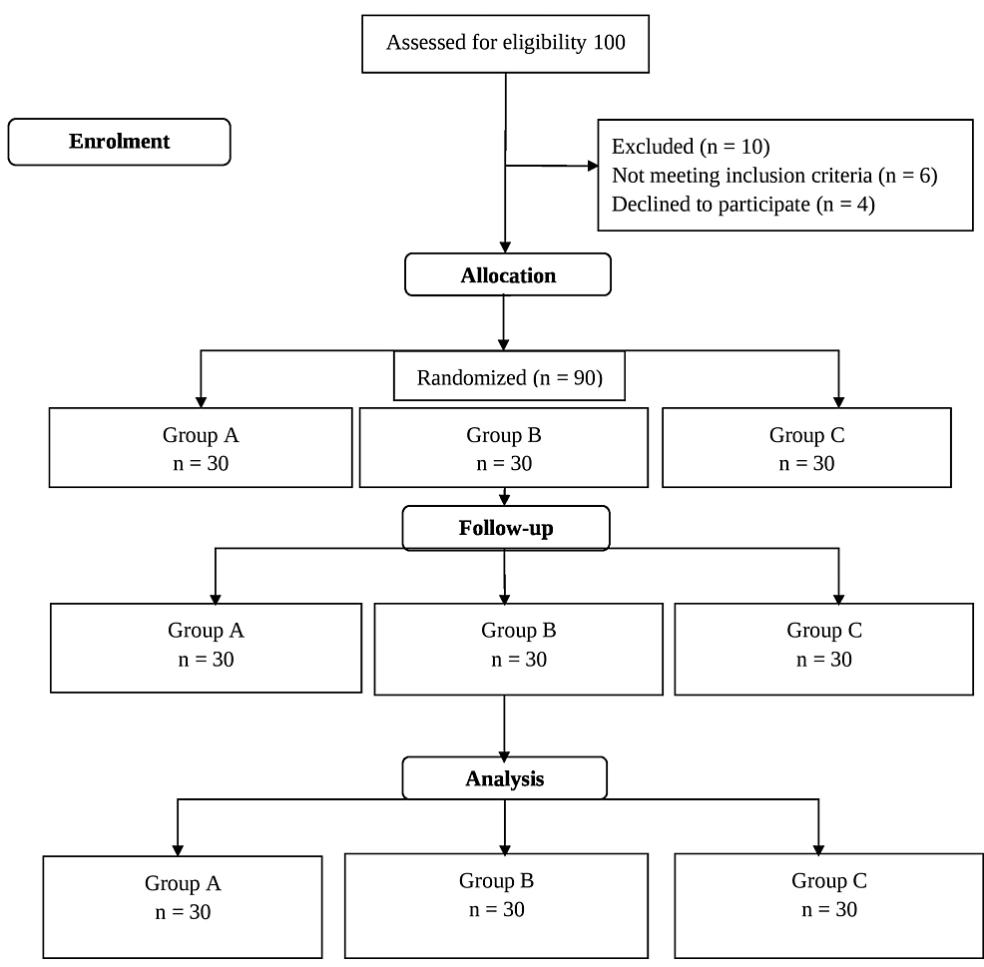
The Consolidated Standards of Reporting Trials (CONSORT) flow chart

A total of 90 patients meeting the inclusion and exclusion criteria were enrolled in the study. All the patients were analyzed, and there was no loss to follow-up (Figure 1).

The distribution of patients entrenched in age, sex, BMI, and ASA physical status was comparable among the three groups. The duration of surgery was also insignificant between the groups (Table 1).

**Table 1:** Demographic characteristics Values are in mean ± standard deviation (SD) or number of patients; *p is not significant; ASA PS: American Society of Anesthesiologists physical status

Parameters	Group A	Group B	Group C	p-value
Age (years)	39.56 ± 10.77	39.5 ± 10.8	33.6 ± 10.58	0.052*
Sex (Male/Female)	16/14	15/15	11/19	0.391*
ASA PS I/II	9/21	8/22	12/18	0.516*
Duration of Surgery (in minutes)	91.83 ± 17.24	85.93 ± 15.21	90.26 ± 8.65	0.254*

All the surgeries were successfully done under laparoscopy with the set flow rate, and no patients needed conversion to different flow rates or open procedures.

There was no significant difference in mean arterial pressure until 30 min after the creation of pneumoperitoneum among the three groups. The baseline MAP was 85.76± 10.11 in group A, 86.03± 9.79 in group B, and 88.13± 8.46 in group C. At 60 min from the creation of the pneumoperitoneum, the MAP increased significantly from 99.17 ± 9.35 in group A, 102.43 ± 8.24 in group B, and 106.83 ± 8.31 in group C. This was statistically significant with a p-value of 0.004. The MAP was also significantly increased at the end of surgery (p-value = 0.004) and 15 min following extubation (p-value = 0.027). The intergroup comparison revealed a significant increase in MAP in group C compared with group A 60 minutes after pneumoperitoneum. The results are summarized in Table 2.

**Table 2:** Changes in the mean arterial pressure (MAP) Values are in mean ± standard deviation (SD) or the number of patients; * p-value not significant; Ϯ p-value significant

Timeline	Group A	Group B	Group C	p-value	Group A vs. Group B	Group A vs.Group C	Group B vs. Group C
T0	83.23± 9.96	84.46± 10.21	83.56± 9.09	0.88O*	0.876*	0.990*	0.932*
T1	85.76± 10.11	86.03± 9.79	88.13± 8.46	0.573*	0.993*	0.599*	0.668*
T2	93.6± 7.33	90.5± 10.05	90.13± 9.29	0.263*	0.377*	0.296*	0.986*
T3	91.67± 10.82	93.3± 10.57	96.6± 8.07	0.151*	0.7997*	0.137*	0.404*
T4	95.96± 9.94	96.93± 9.47	99.3± 7.05	0.332*	0.907*	0.319*	0.560*
T5	100.03± 11.55	100.53± 10.37	102.6± 8.15	0.583*	0.980*	0.589*	0.709*
T6	99.17± 9.35	102.43± 8.24	106.83± 8.31	0.004 Ϯ	0.3152*	0.003 Ϯ	0.126*
T7	97.86± 9.37	99.76± 7.31	104.3± 8.79	0.004 Ϯ	0.133*	0.003 Ϯ	0.302*
T8	99.23± 9.30	102.26± 8.12	105.63± ±8.79	0.022^*^	0.377*	0.016 Ϯ	0.299*
T9	99.7± 12.39	104.16± 10.71	107.5± 9.80	0.027 Ϯ	0.3765*	0.016*	0.299*

The baseline heart rate and the heart rate at the beginning of pneumoperitoneum were similar between the groups. At 10 minutes after pneumoperitoneum, the heart rate increased from 86.7 ± 10.02 in group A, 91.5 ± 11.43 in group B, to 98.03 ± 8.79 in group C (p-value = 0.0002). At 20 minutes following pneumoperitoneum, the heart rate was 88.46 ± 10.75 in group A, 93.53 ± 12.28 in group B, and 100.56 ± 6.82 in group C (p-value < 0.0001). There was a significant increase in heart rate five minutes after arrival in the recovery room (p-value = 0.003). In other timelines (Table 3), there was no significant difference in heart rate among the groups.

**Table 3:** Changes in the heart rate Values are in mean ± standard deviation (SD) or the number of patients; *p- value not significant; Ϯ p-value significant.

Timeline	Group A	Group B	Group C	p-value	Group A vs. Group B	Group A vs. Group C	Group B vs. Group C
T0	85.1 ± 10.49	83.03 ± 12.20	86.73 ± 10.71	0.440*	0.753*	0.839*	0.408*
T1	86.56 ± 12.91	86.2 ± 12.20	89.03 ± 11.55	0.623*	0.992*	0.715*	0.644*
T2	91.16 ± 8.27	92.86 ± 10.92	96.03 ± 8.68	0.130*	0.762*	0.114*	0.392*
T3	86.7 ± 10.02	91.5 ± 11.43	98.03 ± 8.79	0.0002 Ϯ	0.164*	<0.0001 Ϯ	0.0382 Ϯ
T4	88.46 ± 10.75	93.53 ± 12.28	100.56 ± 6.82	<0.0001 Ϯ	0.1384*	< 0.0001 Ϯ	0.0246 Ϯ
T5	92.56 ± 12.72	93.57 ± 12.52	97.33 ± 8.93	0.245*	0.939*	0.250*	0.420*
T6	92.1 ± 10.27	91.1 ± 9.45	95.23 ± 8.04	0.206*	0.909*	0.397*	0.204*
T7	88.43 ± 9.85	88.84 ± 9.46	94.36 ± 7.67	0.021*	0.983*	0.034*	0.052*
T8	87.07±7.72	92.77±10.93	95.23±8.19	0.003 Ϯ	0.044*	0.002 Ϯ	0.547*
T9	99.7± 12.39	104.16± 10.71	107.5± 9.80	0.027 Ϯ	0.3765*	0.016*	0.299*

The nitroglycerin usage was higher in group C (63.3%) than in group B (36.6%) or group A (23.3%). This was statistically significant with a p-value of 0.005. The surgeon satisfaction scores (median, interquartile range) were better in group C (4(5-3)) than in group B (4(5-4)) and group A (4(5-3)). The p-value was 0.009, which was significant statistically. The patient satisfaction scores were higher in group A (4(4-4)) than group B (4(4-3)) and group C (3(4-3)) (p-value < 0.0001).

There was no significant difference in shoulder pain until 16 hours after surgery. The shoulder pain scores were better in group A than in groups B and C after 16 hours (Table 4).

**Table 4:** Postoperative shoulder pain: VAS scores Values are in the median and interquartile range; *p- value not significant; Ϯ p-value significant; VAS – visual analog scale

Time (in hours)	Group A	Group B	Group C	p-value
4	0(0-0)	0(1-0)	0(0.25-0)	0.903*
8	0(0-0)	0(0.25-0)	0(1-0)	0.788*
12	0(0-0)	0(1-0)	0(1-0)	0.398*
16	0(0-0)	0(1-0)	0(1-0)	0.125*
20	0(0-0)	0.5(1-0)	1(1-0)	0.013 Ϯ
24	0(0-0)	0.5(1-0)	1(1-0)	0.011 Ϯ

The surgical site pain scores were also higher in group A than in groups B and C during the first 12 hours postoperatively (Table 5).

**Table 5:** Surgical site pain: VAS score Values are in median and quartile; *p-value not significant; Ϯ p-value significant

Time (in hours)	Group A	Group B	Group C	p-value
4	4(4-3)	3(4-2)	6(4-2)	0.005 Ϯ
8	4(4-3)	3(4-2)	6(4-3)	<0.00001 Ϯ
12	3(4-3)	3(4-2)	7(4-3)	0.011 Ϯ
16	3(4-3)	4(4-3)	8(4-3)	0.092*
20	3(4-3)	4(4-3)	8(4-3)	0.074*
24	3(4-3)	4(4-3)	10(4-3)	0.109*

No complications like bradycardia, hypotension, pneumoperitoneum or surgical bleeding were encountered during the study.

## Discussion

Our study shows that the use of low flow during CO2 insufflation resulted in less severe hemodynamic changes than higher flow rates. The postoperative pain scores were also better with low-flow CO2 insufflation, resulting in better patient satisfaction. The surgeon's satisfaction was better when a higher flow rate was used due to easier operating conditions.

The creation of pneumoperitoneum using CO2 during laparoscopic surgeries has resulted in severe cardiovascular adverse effects, including bradycardia, arrhythmias, or even cardiac arrest [10, 11]. The rapid peritoneal insufflation due to CO2 insufflation may trigger vagal stimulation, resulting in these adverse effects [12]. Several studies have been published about the use of reduced intraperitoneal pressure causing fewer hemodynamic changes during laparoscopic surgeries [13-16]. However, we could not find any studies studying the effects of different CO2 insufflations on hemodynamic changes and postoperative pain scores.

The mean arterial pressure (MAP) increased after CO2 insufflation in all three groups. This increase in MAP was greater in group C than in groups A and B, to a greater extent after 30 minutes. The number of patients requiring nitroglycerin usage due to increased MAP was significantly higher in group C in our study. Since we used nitroglycerin in our study, MAP did not increase much. There was a significant increase in heart rate following CO2 insufflation in our study. Hsu et al. reported four cases (17.5%) of bradycardia following CO2 insufflation at 10 L/min. We did not face any bradycardia in any of the study groups as we used glycopyrrolate in all the patients [17].

The shoulder pain and port site pain were better when low-flow insufflation was used. Hsu et al. showed that a low flow rate of 1 L/min was associated with reduced severity of pain compared to a high flow rate of 10 L/min [17]. The strategy employed by them was to create a pneumoperitoneum with a low flow rate and maintain pressure with a high flow rate. Donatsky et al., in their systematic review of 31 articles, observed that low-pressure pneumoperitoneum minimizes the incidence and severity of shoulder pain [18].

Limitations 

We performed this study without any randomized controlled trials (RCTs) to compare. The fact that all the surgeries were completed with low-flow rate insufflation was a major strength of our study. There are a few limitations to our study. All the surgeries were performed by a single experienced surgeon. An inexperienced surgeon may find it difficult to manage, especially with surgically difficult cases. Secondly, we included only laparoscopic cholecystectomy in our surgery. The reverse Trendelenburg position used in all the patients may have prevented a steep increase in blood pressure. If Trendelenburg positioning is required, as in laparoscopic appendicectomy, the changes in blood pressure may be more pronounced. Finally, though the surgeon was blinded to the groups involved, the difference between different CO2 insufflation flow rates would have been appreciated by the surgeon. This would have affected the surgeon's satisfaction scores.

## Conclusions

The rate of CO2 insufflation during the creation of the pneumoperitoneum in laparoscopic surgeries affects the hemodynamic parameters and postoperative pain. In patients with higher CO2 insufflation, the MAP was significantly increased 60 min after the creation of the pneumoperitoneum and until the end of surgery. The increases in heart rate were significant at 10 and 20 minutes following pneumoperitoneum creation with a higher flow rate.

The patients in group C encountered more severe shoulder pain and surgical sites than the other two groups. The patient satisfaction scores were better with low-flow CO2 insufflation. But surgeon satisfaction was better with higher flow rates. We conclude that low-flow CO2 insufflation during laparoscopic surgeries is associated with fewer hemodynamic changes, lower postoperative port site pain and shoulder pain scores, and better patient satisfaction scores than increased insufflation flow rate.
